# Supplementary material for: Drug repurposing for aging research using model organisms
Source: Aging Cell. 2017 Jun 16;16(5):1006–15. doi: 10.1111/acel.12626 (PMC5595691; doi:10.1111/acel.12626)
Supplement: Supplementary file 7 — Data S1 Zip‐Archive of all report cards. [file ACEL-16-1006-s007.zip › RC_STI.pdf]

STI

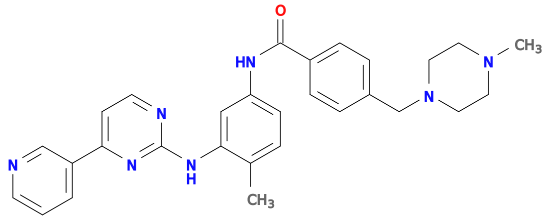

Database identifiers

ChEMBLCompound	CHEMBL941
DrugBank	DB00619
CHEBI	45783
ZINC	ZINC19632618
eMolecules	876446

Ranking

	Rank	Score
Drosophila	6/697	0.95
C. elegans	1/591	1.0

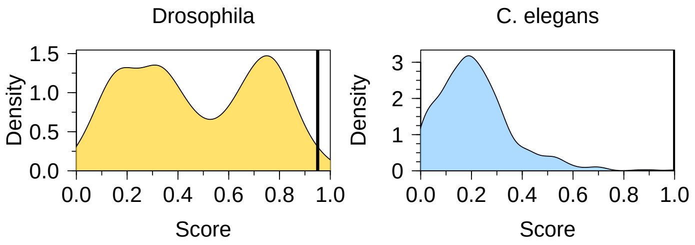

	Ageing implication	Domain conservation	Binding site conservation	Binding affinity	Bioavailability	Lipinski	Promiscuity	Purchasability	Drug approval	**Total**
Drosophila	1.0	0.966	0.985	0.88	(0.9)	0.0	-0.004	0.1	0.1	0.95
C. elegans	1.0	0.96	0.986	0.88	0.969	0.0	-0.004	0.1	0.1	1.0

Names

- **imatinib**
- 4-(4-methyl-piperazin-1-ylmethyl)-n-4-methyl-3-(4-pyridin-3-yl-pyrimidin-2-ylamino)-phenyl-benzamide
- alpha-(4-methyl-1-piperazinyl)-3'-((4-(3-pyridyl)-2-pyrimidinyl)amino)-p-toluidide
- Gleevec
- Sti 571

Roles

apoptosis inducer, tyrosine kinase inhibitor, antineoplastic agent

Status

Approved drug (according to ChEMBL)	Yes
Administration Route	Oral
Number of Rule of 5 violations	0
Binding affinity to original target in log units (RF-Score prediction)	7.0
Burns *C. elegans* bioavailability prediction	11.24

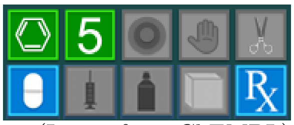

(Image from ChEMBL)

Medical Information

Indicatation: For the treatment of Philadelphia chromosome positive chronic myeloid leukemia (Ph+ CML), Ph+ acute lymphoblastic leukaemia, myelodysplastic/myeloproliferative diseases, aggressive systemic mastocytosis, hypereosinophilic syndrome and/or chronic eosinophilic leukemia (CEL), dermatofibrosarcoma protuberans, and malignant gastrointestinal stromal tumors (GIST).
Mechanism of action: Imatinib mesylate is a protein-tyrosine kinase inhibitor that inhibits the Bcr-Abl tyrosine kinase, the constitutive abnormal tyrosine kinase created by the Philadelphia chromosome abnormality in chronic myeloid leukemia (CML). It inhibits proliferation and induces apoptosis in Bcr-Abl positive cell lines as well as fresh leukemic cells from Philadelphia chromosome positive chronic myeloid leukemia. Imatinib also inhibits the receptor tyrosine kinases for platelet derived growth factor (PDGF) and stem cell factor (SCF) - called c-kit. Imatinib was identified in the late 1990s by Dr Brian J. Druker. Its development is an excellent example of rational drug design. Soon after identification of the bcr-abl target, the search for an inhibitor began. Chemists used a high-throughput screen of chemical libraries to identify the molecule 2-phenylaminopyrimidine. This lead compound was then tested and modified by the introduction of methyl and benzamide groups to give it enhanced binding properties, resulting in imatinib.
Toxicity: The most frequently reported adverse reactions (>30%) were edema, nausea,
Metabolism: Primarily hepatic via CYP3A4. Other cytochrome P450 enzymes, such as CYP1A2, CYP2D6, CYP2C9, and CYP2C19, play a minor role in its metabolism. The main circulating active metabolite in humans is the N-demethylated piperazine derivative, formed predominantly by CYP3A4. This metabolite is similar in potency to the parent compound.
Retail: Murfreesboro Pharmaceutical Nursing Supply, Novartis AG, Physicians Total Care Inc.
Gleevec 100 mg tablet - 41.69 USD / tablet
Gleevec 400 mg tablet - 174.38 USD / tablet

(Information from DrugBank)

Compound Target Characteristics

Tyrosine-protein kinase ABL1

Best gene implication in ageing for this target family came from gene P00519 via mapping the annotation from Ensembl ENSG00000097007 via mapping the annotation from EntrezGene 25 via mapping the annotation from GenAgeHuman 0078 annotated in GenAge release 17.

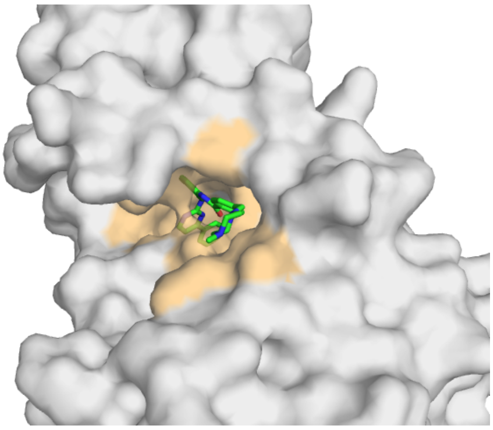

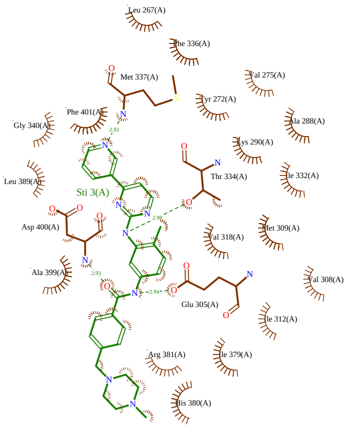

protein	amino acids contacts (binding site)
PDB:3pyy:chainA:P00519	L Y V A K E V M I V I T F M G I H R L A D F
sp:P00519:ABL1_HUMAN	L Y V A K E V M I V I T F M G I H R L A D F
tr:Q59FK4:Q59FK4_HUMAN	L Y V A K E V M I V I T F M G I H R L A D F
tr:F1M0A6:F1M0A6_RAT	L Y V A K E V M I V I T F M G I H R L A D F
tr:D3ZGM3:D3ZGM3_RAT	L Y V A K E V M I V I T F M G I H R L A D F
tr:E9PT20:E9PT20_RAT	L Y V A K E V M I V I T F M G I H R L A D F
tr:Q3V2W1:Q3V2W1_MOUSE	L Y V A K E V M I V I T F M G I H R L A D F
sp:P00520:ABL1_MOUSE	L Y V A K E V M I V I T F M G I H R L A D F
tr:Q3SYK5:Q3SYK5_MOUSE	L Y V A K E V M I V I T F M G I H R L A D F
sp:P00522:ABL_DROME	L Y V A K E I M M V I T F M G I H R L A D F
tr:A8JNU2:A8JNU2_DROME	L Y V A K E I M M V I T F M G I H R L A D F
sp:P03949:ABL1_CAEEL	L Y V A K E I M L V I T F M G I H R L A D F

	whole protein		domain-based		contact-based	
protein	ident	simil	ident	simil	ident	simil
PDB:3pyy:chainA:P00519	1.0	1.0	1.0	1.0	1.0	1.0
sp:P00519:ABL1_HUMAN	1.0	1.0	1.0	1.0	1.0	1.0
tr:Q59FK4:Q59FK4_HUMAN	0.95	0.96	1.0	1.0	1.0	1.0
tr:F1M0A6:F1M0A6_RAT	0.84	0.93	1.0	1.0	1.0	1.0
tr:D3ZGM3:D3ZGM3_RAT	0.87	0.95	1.0	1.0	1.0	1.0
tr:E9PT20:E9PT20_RAT	0.84	0.93	1.0	1.0	1.0	1.0
tr:Q3V2W1:Q3V2W1_MOUSE	0.3	0.32	1.0	1.0	1.0	1.0
sp:P00520:ABL1_MOUSE	0.87	0.95	1.0	1.0	1.0	1.0
tr:Q3SYK5:Q3SYK5_MOUSE	0.84	0.93	1.0	1.0	1.0	1.0
sp:P00522:ABL_DROME	0.26	0.5	0.78	0.93	0.91	0.98
tr:A8JNU2:A8JNU2_DROME	0.26	0.5	0.78	0.93	0.91	0.98
sp:P03949:ABL1_CAEEL	0.32	0.64	0.67	0.9	0.91	0.99

Abl (FBgn0000017) associated phenotypes

cell migration defective, cell shape defective, germline clone, heat sensitive, lethal - all die before end of pupal stage, maternal effect, mitotic cell cycle defective, neuroanatomy defective, neurophysiology defective, partially lethal - majority die, partially lethal - majority live, reduced, rescuable maternal effect, size defective, somatic clone, some die during pupal stage

(Information from FlyBase)

Abl (UniProt:P00522) annotation

Function: Arm and Abl proteins function cooperatively at adherens junctions in both the CNS and epidermis; critical for embryonic epithelial morphogenesis regulating cell shape changes and cell migration. Plays a critical role in transducing embryonic midline repulsive cues; may regulate cytoskeletal dynamics underlying a growth cone's response to midline cues. The ability of pCC/MP2 axons to correctly interpret midline repulsive cues and stay on the ipsilateral side is dependent on the strength of both Slit/robo and Abl-dependent signaling pathways. (PubMed:11756472, PubMed:12973825, PubMed:9635189).
Subcellular location: Cytoplasm.
Tissue specificity: Arm and ena colocalize with Abl at adherens junctions throughout development. (PubMed:11756472, PubMed:9635189).
Developmental stage: Expressed both maternally and zygotically. (PubMed:2832740).
Disruption phenotype: Both loss- and gain-of-function mutants exhibit neurons within the pCC/MP2 pathway that incorrectly project across the midline. Loss of Abl disrupts cell migration and cell shape changes during dorsal closure. (PubMed:11756472, PubMed:9635189).

(Information from UniProt)

abl-1 (WBGene00000018) associated phenotypes

apoptosis variant, gamma ray induced apoptosis increased, lipid synthesis increased, protein subcellular localization variant

(Information from WormBase)

Tyrosine-protein kinase ABL1

Best gene implication in ageing for this target family came from gene P00519 via mapping the annotation from Ensembl ENSG00000097007 via mapping the annotation from EntrezGene 25 via mapping the annotation from GenAgeHuman 0078 annotated in GenAge release 17.

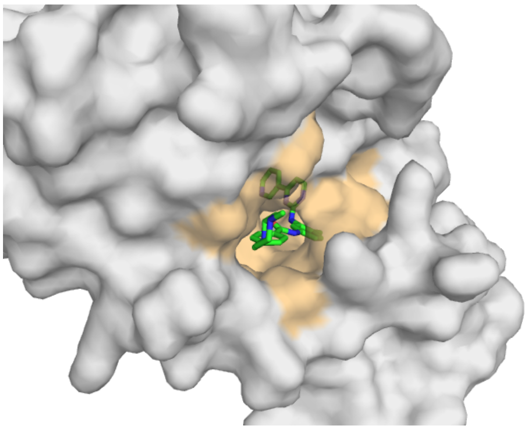

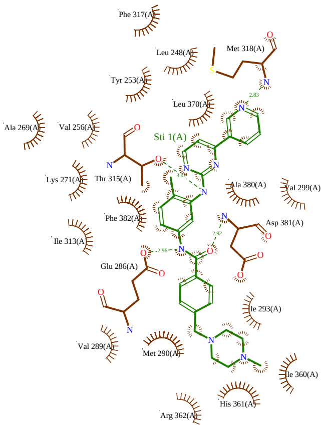

protein	amino acids contacts (binding site)																					
PDB:1iep:chainA:P00520	L	Y	V	A	K	E	V	M	I	V	I	T	F	M	G	I	H	R	L	A	D	F
PDB:1opj:chainA:P00520	L	Y	V	A	K	E	V	M	I	V	I	T	F	M	G	I	H	R	L	A	D	F
PDB:3k5v:chainA:P00520	L	Y	V	A	K	E	V	M	I	V	I	T	F	M	G	I	H	R	L	A	D	F
PDB:3ms9:chainA:P00520	L	Y	V	A	K	E	V	M	I	V	I	T	F	M	G	I	H	R	L	A	D	F
PDB:3mss:chainA:P00520	L	Y	V	A	K	E	V	M	I	V	I	T	F	M	G	I	H	R	L	A	D	F
sp:P00519:ABL1_HUMAN	L	Y	V	A	K	E	V	M	I	V	I	T	F	M	G	I	H	R	L	A	D	F
tr:Q59FK4:Q59FK4_HUMAN	L	Y	V	A	K	E	V	M	I	V	I	T	F	M	G	I	H	R	L	A	D	F
tr:F1M0A6:F1M0A6_RAT	L	Y	V	A	K	E	V	M	I	V	I	T	F	M	G	I	H	R	L	A	D	F
tr:D3ZGM3:D3ZGM3_RAT	L	Y	V	A	K	E	V	M	I	V	I	T	F	M	G	I	H	R	L	A	D	F
tr:E9PT20:E9PT20_RAT	L	Y	V	A	K	E	V	M	I	V	I	T	F	M	G	I	H	R	L	A	D	F
tr:Q3V2W1:Q3V2W1_MOUSE	L	Y	V	A	K	E	V	M	I	V	I	T	F	M	G	I	H	R	L	A	D	F
sp:P00520:ABL1_MOUSE	L	Y	V	A	K	E	V	M	I	V	I	T	F	M	G	I	H	R	L	A	D	F
tr:Q3SYK5:Q3SYK5_MOUSE	L	Y	V	A	K	E	V	M	I	V	I	T	F	M	G	I	H	R	L	A	D	F
sp:P00522:ABL_DROME	L	Y	V	A	K	E	I	M	M	V	I	T	F	M	G	I	H	R	L	A	D	F
tr:A8JNU2:A8JNU2_DROME	L	Y	V	A	K	E	I	M	M	V	I	T	F	M	G	I	H	R	L	A	D	F
sp:P03949:ABL1_CAEEL	L	Y	V	A	K	E	I	M	L	V	I	T	F	M	G	I	H	R	L	A	D	F

	whole protein		domain-based		contact-based	
protein	ident	simil	ident	simil	ident	simil
PDB:1iep:chainA:P00520	0.99	1.0	1.0	1.0	1.0	1.0
PDB:1opj:chainA:P00520	0.99	1.0	1.0	1.0	1.0	1.0
PDB:3k5v:chainA:P00520	0.99	1.0	1.0	1.0	1.0	1.0
PDB:3ms9:chainA:P00520	0.99	1.0	1.0	1.0	1.0	1.0
PDB:3mss:chainA:P00520	0.99	1.0	1.0	1.0	1.0	1.0
sp:P00519:ABL1_HUMAN	0.88	0.95	1.0	1.0	1.0	1.0
tr:Q59FK4:Q59FK4_HUMAN	0.83	0.92	1.0	1.0	1.0	1.0
tr:F1M0A6:F1M0A6_RAT	0.93	0.96	1.0	1.0	1.0	1.0
tr:D3ZGM3:D3ZGM3_RAT	0.96	0.99	1.0	1.0	1.0	1.0
tr:E9PT20:E9PT20_RAT	0.93	0.96	1.0	1.0	1.0	1.0
tr:Q3V2W1:Q3V2W1_MOUSE	0.33	0.33	1.0	1.0	1.0	1.0
sp:P00520:ABL1_MOUSE	1.0	1.0	1.0	1.0	1.0	1.0
tr:Q3SYK5:Q3SYK5_MOUSE	0.96	0.98	1.0	1.0	1.0	1.0
sp:P00522:ABL_DROME	0.26	0.49	0.78	0.93	0.91	0.98
tr:A8JNU2:A8JNU2_DROME	0.26	0.48	0.78	0.93	0.91	0.98
sp:P03949:ABL1_CAEEL	0.31	0.63	0.67	0.9	0.91	0.99

Abl (FBgn0000017) associated phenotypes

cell migration defective, cell shape defective, germline clone, heat sensitive, lethal - all die before end of pupal stage, maternal effect, mitotic cell cycle defective, neuroanatomy defective, neurophysiology defective, partially lethal - majority die, partially lethal - majority live, reduced, rescuable

maternal effect, size defective, somatic clone, some die during pupal stage

(Information from FlyBase)

Abl (UniProt:P00522) annotation

Function: Arm and Abl proteins function cooperatively at adherens junctions in both the CNS and epidermis; critical for embryonic epithelial morphogenesis regulating cell shape changes and cell migration. Plays a critical role in transducing embryonic midline repulsive cues; may regulate cytoskeletal dynamics underlying a growth cone's response to midline cues. The ability of pCC/MP2 axons to correctly interpret midline repulsive cues and stay on the ipsilateral side is dependent on the strength of both Slit/robo and Abl-dependent signaling pathways. (PubMed:11756472, PubMed:12973825, PubMed:9635189).
Subcellular location: Cytoplasm.
Tissue specificity: Arm and ena colocalize with Abl at adherens junctions throughout development. (PubMed:11756472, PubMed:9635189).
Developmental stage: Expressed both maternally and zygotically. (PubMed:2832740).
Disruption phenotype: Both loss- and gain-of-function mutants exhibit neurons within the pCC/MP2 pathway that incorrectly project across the midline. Loss of Abl disrupts cell migration and cell shape changes during dorsal closure. (PubMed:11756472, PubMed:9635189).

(Information from UniProt)

abl-1 (WBGene00000018) associated phenotypes

apoptosis variant, gamma ray induced apoptosis increased, lipid synthesis increased, protein subcellular localization variant

(Information from WormBase)

Mitogen-activated protein kinase 14

Best gene implication in ageing for this target family came from gene O62618 annotated in UniProt release 2014_02. Annotation GO 8340 (determination of adult lifespan) was Inferred from Mutant Phenotype

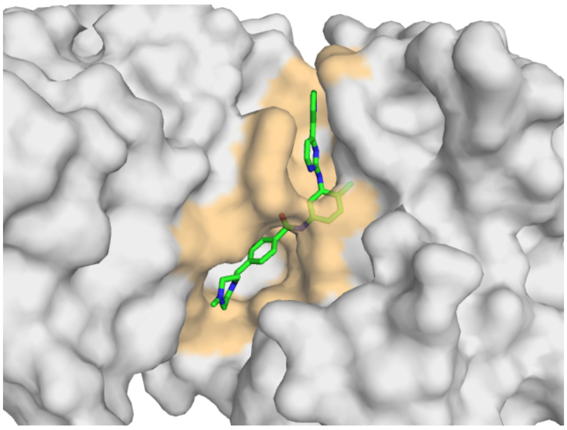

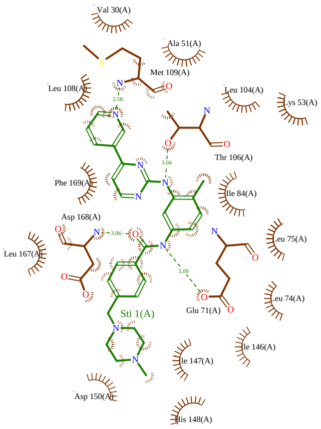

protein	amino acids contacts (binding site)
PDB:3hec:chainA:Q16539	V A K E L L I L T L M I I H D L D F
sp:Q15759:MK11_HUMAN	V A K E L L I L T L M I I H D L D F
tr:L7RSM2:L7RSM2_HUMAN	V A K E L L I L T L M I I H D L D F
sp:Q16539:MK14_HUMAN	V A K E L L I L T L M I I H D L D F
tr:D4A3U7:D4A3U7_RAT	V A K E L L I L T L M I I H D L D F
tr:G3V617:G3V617_RAT	V A K E L L I L T L M I I H D L D F
tr:Q56A33:Q56A33_RAT	V A K E L L I L T L M I I H D L D F
tr:B2KF34:B2KF34_MOUSE	V A K E L L I L T L M I I H D L D F
tr:B2KF35:B2KF35_MOUSE	V A K E L L I L T L M I I H D L D F
sp:P47811:MK14_MOUSE	V A K E L L I L T L M I I H D L D F
sp:Q9WUI1:MK11_MOUSE	V A K E L L I L T L M I I H D L D F
tr:Q5U421:Q5U421_MOUSE	V A K E L L I L T L M I I H D L D F
sp:O62618:MK14A_DROME	V A K E L L I L T L M V I H D L D F
tr:E1JIV6:E1JIV6_DROME	V A K E L L I L T L M V I H D L D F
sp:Q17446:PMK1_CAEEL	I A K E L L I F S L M I I H D L D F
sp:P32485:HOG1_YEAST	V A K E L L I F T L Q V I H D C D F

	whole protein		domain-based		contact-based	
protein	ident	simil	ident	simil	ident	simil
PDB:3hec:chainA:Q16539	1.0	1.0	1.0	1.0	1.0	1.0
sp:Q15759:MK11_HUMAN	0.73	0.92	0.78	0.94	1.0	1.0
tr:L7RSM2:L7RSM2_HUMAN	0.96	0.99	0.95	0.99	1.0	1.0
sp:Q16539:MK14_HUMAN	1.0	1.0	1.0	1.0	1.0	1.0
tr:D4A3U7:D4A3U7_RAT	0.74	0.92	0.79	0.95	1.0	1.0
tr:G3V617:G3V617_RAT	0.99	1.0	0.99	1.0	1.0	1.0
tr:Q56A33:Q56A33_RAT	0.95	0.99	0.95	0.99	1.0	1.0
tr:B2KF34:B2KF34_MOUSE	0.56	0.56	0.62	0.62	1.0	1.0
tr:B2KF35:B2KF35_MOUSE	0.7	0.71	0.8	0.8	1.0	1.0
sp:P47811:MK14_MOUSE	0.99	1.0	0.99	1.0	1.0	1.0
sp:Q9WUI1:MK11_MOUSE	0.74	0.92	0.78	0.95	1.0	1.0
tr:Q5U421:Q5U421_MOUSE	0.96	0.99	0.95	0.99	1.0	1.0
sp:O62618:MK14A_DROME	0.67	0.88	0.74	0.91	0.94	0.98
tr:E1JIV6:E1JIV6_DROME	0.67	0.88	0.74	0.91	0.94	0.98
sp:Q17446:PMK1_CAEEL	0.62	0.85	0.71	0.92	0.83	0.94
sp:P32485:HOG1_YEAST	0.41	0.68	0.54	0.85	0.78	0.82

Mpk2 (FBgn0015765) associated phenotypes

heat stress response defective, immune response defective, non-enhancer of variegation, non-suppressor of variegation, nutrition conditional, osmotic stress response defective, oxidative stress response defective, partially lethal - majority die, reduced, short lived, starvation stress response defective, stress response defective

(Information from FlyBase)

Mpk2 (UniProt:O62618) annotation

Function: Kinase involved in a signal transduction pathway. May down-regulate insect immunity gene expression after prolonged infection. (PubMed:9417090, PubMed:9584193).
Cofactor:Mg(2+)
Enzyme regulation: Activated by threonine and tyrosine phosphorylation by Mkk3 in response to environmental stress. (PubMed:9417090, PubMed:9584193).
Subcellular location: Nucleus (PubMed:9417090).
Developmental stage: Expressed both maternally and zygotically. Levels are highest at the preblastoderm stage but low levels are present throughout development. (PubMed:9584193).
Domain: The TXY motif contains the threonine and tyrosine residues whose phosphorylation activates the MAP kinases.
Ptm: Dually phosphorylated on Thr-184 and Tyr-186, which activates the enzyme. (PubMed:9417090).

(Information from UniProt)

pmk-1 (WBGene00004055) associated phenotypes
cell stress response variant, drug induced gene expression variant, gene expression level reduced, lethal, life span variant, organism oxidative stress response hypersensitive, pathogen induced gene expression variant, pathogen susceptibility increased, sterile

(Information from WormBase)

pmk-1 (UniProt:Q17446) annotation
Function: Responds to activation by environmental stress and pro- inflammatory cytokines by phosphorylating downstream targets. Functions downstream of the MAPKK sek-1 and the MAPKKK nsy-1 as the MAP kinase required for pathogen resistance. (PubMed:11703092, PubMed:12142542).
Cofactor: Mg(2+) Evidence=(PubMed:11703092);
Enzyme regulation: Activated by phosphorylation on threonine and tyrosine. Inhibited by pyridinyl-imidazole related compounds. (PubMed:11703092).
Domain: The TXY motif contains the threonine and tyrosine residues whose phosphorylation activates the MAP kinases.
Ptm: Dually phosphorylated on Thr-191 and Tyr-193, which activates the enzyme.

(Information from UniProt)
